# Clinical feasibility of miniaturized Lissajous scanning confocal laser endomicroscopy for indocyanine green-enhanced brain tumor diagnosis

**DOI:** 10.3389/fonc.2022.994054

**Published:** 2023-01-13

**Authors:** Duk Hyun Hong, Jang Hun Kim, Jae-Kyung Won, Hyungsin Kim, Chayeon Kim, Kyung-Jae Park, Kyungmin Hwang, Ki-Hun Jeong, Shin-Hyuk Kang

**Affiliations:** ^1^ Department of Neurosurgery, Korea University Hospital, Korea University College of Medicine, Seoul, Republic of Korea; ^2^ Department of Pathology, Seoul National University Hospital, Seoul National University College of Medicine, Seoul, Republic of Korea; ^3^ VPIX Medical Inc., Daejeon, Republic of Korea; ^4^ Department of Bio and Brain Engineering, KAIST Institute for Health Science and Technology (KIHST), Korea Advanced Institute of Science and Technology (KAIST), Seoul, Republic of Korea

**Keywords:** brain neoplasm, confocal microscopy, Lissajous scanning, indocyanine green, real-time diagnosis

## Abstract

**Background:**

Intraoperative real-time confocal laser endomicroscopy (CLE) is an alternative modality for frozen tissue histology that enables visualization of the cytoarchitecture of living tissues with spatial resolution at the cellular level. We developed a new CLE with a “Lissajous scanning pattern” and conducted a study to identify its feasibility for fluorescence-guided brain tumor diagnosis.

**Materials and methods:**

Conventional hematoxylin and eosin (H&E) histological images were compared with indocyanine green (ICG)-enhanced CLE images in two settings (1): experimental study with in vitro tumor cells and ex vivo glial tumors of mice, and (2) clinical evaluation with surgically resected human brain tumors. First, CLE images were obtained from cultured U87 and GL261 glioma cells. Then, U87 and GL261 tumor cells were implanted into the mouse brain, and H&E staining was compared with CLE images of normal and tumor tissues *ex vivo*. To determine the invasion of the normal brain, two types of patient-derived glioma cells (CSC2 and X01) were used for orthotopic intracranial tumor formation and compared using two methods (CLE vs. H&E staining). Second, in human brain tumors, tissue specimens from 69 patients were prospectively obtained after elective surgical resection and were also compared using two methods, namely, CLE and H&E staining. The comparison was performed by an experienced neuropathologist.

**Results:**

When ICG was incubated *in vitro*, U87 and GL261 cell morphologies were well-defined in the CLE images and depended on dimethyl sulfoxide. *Ex vivo* examination of xenograft glioma tissues revealed dense and heterogeneous glioma cell cores and peritumoral necrosis using both methods. CLE images also detected invasive tumor cell clusters in the normal brain of the patient-derived glioma xenograft model, which corresponded to H&E staining. In human tissue specimens, CLE images effectively visualized the cytoarchitecture of the normal brain and tumors. In addition, pathognomonic microstructures according to tumor subtype were also clearly observed. Interestingly, in gliomas, the cellularity of the tumor and the density of streak-like patterns were significantly associated with tumor grade in the CLE images. Finally, panoramic view reconstruction was successfully conducted for visualizing a gross tissue morphology.

**Conclusion:**

In conclusion, the newly developed CLE with Lissajous laser scanning can be a helpful intraoperative device for the diagnosis, detection of tumor-free margins, and maximal safe resection of brain tumors.

## 1 Introduction

Resection of brain tumors improves patient conditions, including reduced mass effect on the brain, prevention of tumor recurrence, and increased patient survival (1–4). However, total tumor removal is not always possible, and increasing the resection area could result in a permanent neurological deficit because it is not easy to determine the normal brain tissue at the tumor interface during a surgical operation. With technological advances, neuronavigation systems and 5-aminolevulinic acid (5-ALA) fluorescence dyes have improved surgical outcomes in tumor resection (5, 6). However, these tools remain insufficient because of the brain shifts and low fluorescence specificity (7). Therefore, direct histological visualization *via* multiple optical biopsies could be performed for proper intraoperative diagnosis, identification of existing remnant tumors, and increased tumor resection (8).

Thus far, the classical tool for intraoperative brain tumor detection has been to obtain frozen section examination. To identify the histological diagnosis and tumor margins, tissue samples were delivered to a pathologist, who then performed frozen tissue preparation for rapid interpretation. This process has several limitations (9, 10). Usually, it requires up to 20 min or longer per sample for diagnosis, thus increasing the operation time (11), and a longer surgical time can cause postoperative complications in cases of multiple tissue examinations (12). In addition, proper diagnosis cannot be achieved in a small tissue sample. Moreover, mechanical tissue disruption due to tumor removal is a non-diagnostic or misleading problem in frozen tissue diagnosis (13). Examination of normal tissue and tumor interfaces sometimes causes irreversible neurological deficits (14). Therefore, the surgical room still requires a more rapid and efficient method to properly diagnose tumors and improve surgical outcomes in patients with gliomas and other brain tumors. It would be greatly advantageous to develop a minimally invasive, real-time, intraoperative tissue diagnostic method.

Intraoperative confocal laser endomicroscopy (CLE) is a novel technology that enables the visualization of living tissue cytoarchitecture with cellular level spatial resolution (15). It was developed for microscopic miniaturization with a handheld probe and for the slide-free imaging of thin planes within the whole tissue. Therefore, minimized CLE can be used to examine freshly excised tissue from patients after staining with biocompatible dyes, such as fluorescein and indocyanine green (ICG). Previous studies have reported the clinical feasibility of diagnosis in various cancers, including gastrointestinal and lung cancers (16, 17). In the field of brain tumor surgery, CLE has been reported to be suitable for clinical applications (18–20). Recently, we developed a handheld CLE in the near-infrared band that is capable of ICG imaging (21, 22). This product can acquire fast and clear images using an ultracompact laser scanner that implements a Lissajous scanning pattern. We acquired *ex vivo* images of various extracted mouse organs, such as the lungs, kidneys, and bladder, from ICG-injected mice without the usual preparation of samples and showed the distinct structures of each organ. These data can be used to evaluate various brain tumor scenarios.

In this study, we examined cell morphology *in vitro* and tissue microstructures *ex vivo* in two glioma cell lines. In addition, tumor invasion into normal tissue was identified in two patient-derived xenograft models using Lissajous scanning CLE. To further evaluate clinical applications for real-time diagnosis, we used freshly resected specimens of various brain tumors for *ex vivo* direct CLE imaging. Hematoxylin and eosin (H&E)-stained images served as the gold standard in all models and were compared with CLE images.

## 2 Materials and methods

### 2.1 Patient enrollment and ethics statement

**Figure 1:**
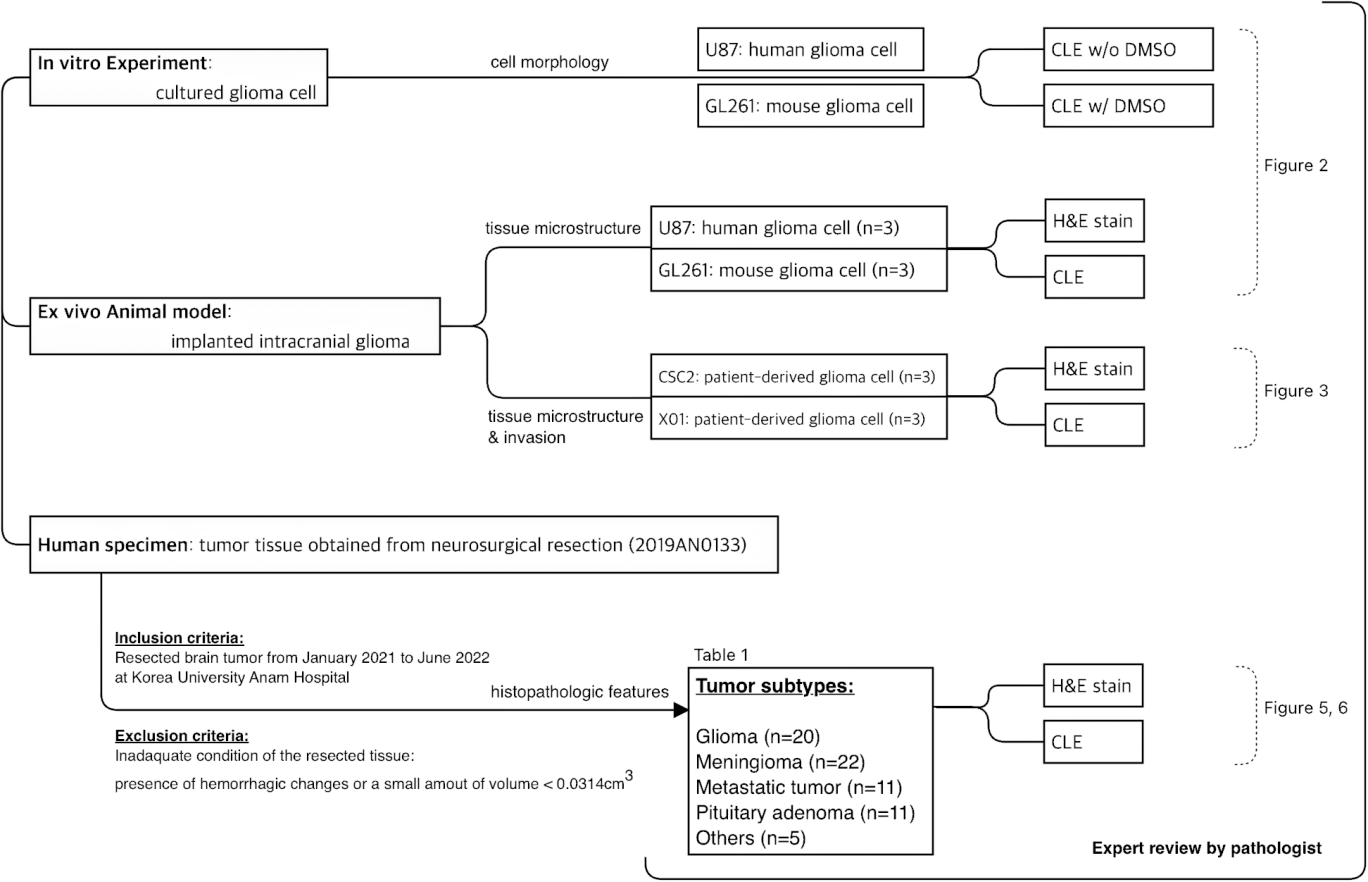
The flowchart of the study.

Patients with tumorous conditions in the brain that required surgical resection from January 2021 to June 2022 were included in the study. They consisted of various types of brain tumors such as meningiomas, astrocytomas, pituitary adenomas, and metastatic tumors. All experiments using human tissues were performed with the approval of the Institutional Review Board of Korea University Anam Hospital in strict accordance with the Code of Ethics of the World Medical Association for experiments (approval number: 2019AN0133). Before elective brain tumor surgery, voluntary informed consent was obtained from the patients and their legal guardians after they were fully informed about the study design and that the experiments were conducted using *ex vivo* methods, which is not be potentially harmful to the patients. Patients were excluded if the resected tissue was inadequate for examination by CLE, such as presence of hemorrhagic changes or a small amount of volume <0.0314cm^3^ (suspected volume of one-piece tumor tissue *via* stereotactic navigation biopsy system, StealthStation S8, Medtronic, Minneapolis, MN, USA). The flowchart of the study was presented in **Figure 1**.

### 2.2 Cell culture

The GL261 mouse glioma cell line and U87 human glioma cell line were cultured in high-glucose Dulbecco’s modified Eagle’s medium (DMEM; Welgene, Gyeongsan, Republic of Korea) supplemented with 10% fetal bovine serum (Welgene), 100 U/mL penicillin, and 0.1 mg/mL streptomycin in a monolayer culture at 37°C in a humidified atmosphere containing 5% CO_2_. Patient-derived glioma stem-like cells, CSC2 and X01, were cultured in a proliferation medium composed of DMEM/F-12, B-27 supplement (Gibco, Waltham, MA, USA), 10 ng/mL recombinant human bFGF (PeproTech, Cranbury, NJ, USA), 20 ng/mL recombinant human EGF (R&D Systems, Minneapolis, MN, USA), 20 U/mL penicillin, and 20 μg/mL streptomycin at 37°C in a humidified atmosphere containing 5% CO_2_.

### 2.3 Tumor implantation

All procedures were conducted in accordance with the guidelines and protocols approved by the Institutional Animal Care and Use Committee of the Korea University College of Medicine (approval number: KOREA-2020-0079). Five- to six-week-old BALB/c nude and C57BL/6 mice were purchased from Orient Bio (Seongnam, Republic of Korea). For intracranial implantation, animals were anesthetized with ketamine (100 mg/kg) and xylazine (10 mg/kg). A total of 1 × 10^5^ cells in 3 µL of Hanks’ balanced salt solution were injected into the brain at a rate of 1 µL/min using a Hamilton syringe controlled by a stereotaxic device (David Kopf Instruments, Tujunga, CA). Coordinates for intracranial inoculation were −0.2 mm anteroposterior, +2.2 mm mediolateral, and −3.5 mm dorsoventral from the bregma.

### 2.4 Imaging device

A CLE system (cCeLL ex vivo; VPIX Medical, Daejeon, Republic of Korea) was used for histoarchitectural imaging of slide-free whole tissues stained with a fluorescent dye. The cCeLL ex vivo image was based on a 785-nm laser system with a near-infrared filter to detect emissions from 800–860 nm. A microscope head and probe (PixectionTM, VPIX Medical) with an outer diameter of 4 mm directly contacted the patient’s tissue and irradiated the tissue with a Lissajous laser-scanning pattern to acquire a tissue image within 100 µm from the surface. The probe scanned patient tissues with 10 frame rates at a field of view (FOV) of 300 µm × 300 µm, corresponding to approximately 660x magnification. The system reconstructed the acquired data as high-quality images of 1024 × 1024 pixels that were saved as images or videos. The probe was also used with a dedicated probe stage for fine movement in the XYZ direction.

### 2.5 ICG treatment and image acquisition

For image acquisition both *in vitro* and *ex vivo*, ICG (Sigma-Aldrich, St. Louis, MO) fluorescence was used. ICG solvent dissolved in 30% EtOH at a concentration of 0.5 mg/mL was utilized for labelling both cell lines and patient tissue samples. U87MG and GL261 cells were seeded at 5 × 10^4^ cells/well in 12-well plates to 70% confluence on top of sterile glass coverslips. The medium was removed and the cells were washed three times with phosphate-buffered saline (PBS) for 5 min. Cells grown on coverslips were incubated in ICG with or without 10% dimethyl sulfoxide (DMSO) and imaged using CLE.

The mouse brain image acquisition was utilized according to the following methods: intravenous tail injection of ICG before mouse sacrifice or *ex vivo* incubation of the mouse brain into the ICG solution. Intravenously administered mice were injected with 100 µL of ICG solution (0.5 mg/mL) and left for 5 min to allow the full diffusion of ICG. Thereafter, the brains were removed, and a coronal section of the sample was placed at a thickness of 1 cm before CLE was conducted. Similarly, in the ICG incubation group, the tissues were sectioned and placed in a Petri dish for incubation following brain removal. Brain tissue was incubated with 100 µL of ICG solution (0.5 mg/mL) prior to CLE imaging. Excess ICG solution left on the imaging surface of the sample was cleaned with a tissue wiper for optimal image acquisition.

The method used to image the tumor tissues was identical to that used for the mouse brain preparations. Tumor specimen was obtained in the center of the resected tumor and sectioned at a thickness of 1 cm before incubation with 100 µL of ICG solution (0.5 mg/mL) for 5 min. Following incubation, the tissues were cleaned with a tissue wiper to remove excess ICG before imaging with CLE.

### 2.6 H&E and immunofluorescence staining

After the CLE imaging experiments, tumor tissues corresponding to the imaging site were resected and fixed in 4% formalin. Formalin-fixed and paraffin-embedded sections (4 μm thickness) were prepared for H&E staining. Immunofluorescence (IF) staining was performed on sections fixed in cold methanol for 10 min at -20°C, rinsed in PBS, incubated with primary antibodies, and diluted in PBS containing 1% bovine serum albumin (BSA) and 0.25% Triton X-100 overnight at 4°C. Antibodies against myelin basic protein (MBP, ab40390; Abcam, Cambridge, UK), glial fibrillary acidic protein (z0334; Dako, Glostrup, Denmark), and neurofilament-M (2H3; DSHB, Iowa City, IA) were used. Slides were then incubated with fluorescence-conjugated secondary antibodies and mounted with Vectashield^®^ Hard Set™ mounting medium (Vector Laboratories, Newark, CA) containing 4’,6-diamidino-2-phenylindole (DAPI). H&E and immunostained slices were observed using light and fluorescence microscopy (Carl Zeiss, Oberkochen, Germany) and compared with CLE images obtained by an experienced pathologist (JKW).

## 3 Results

### 3.1 *In vitro* and *ex vivo* glial tumors of mice

#### 3.1.1 CLE imaging of glial tumor cells and orthotopic tumors

**Figure 2:**
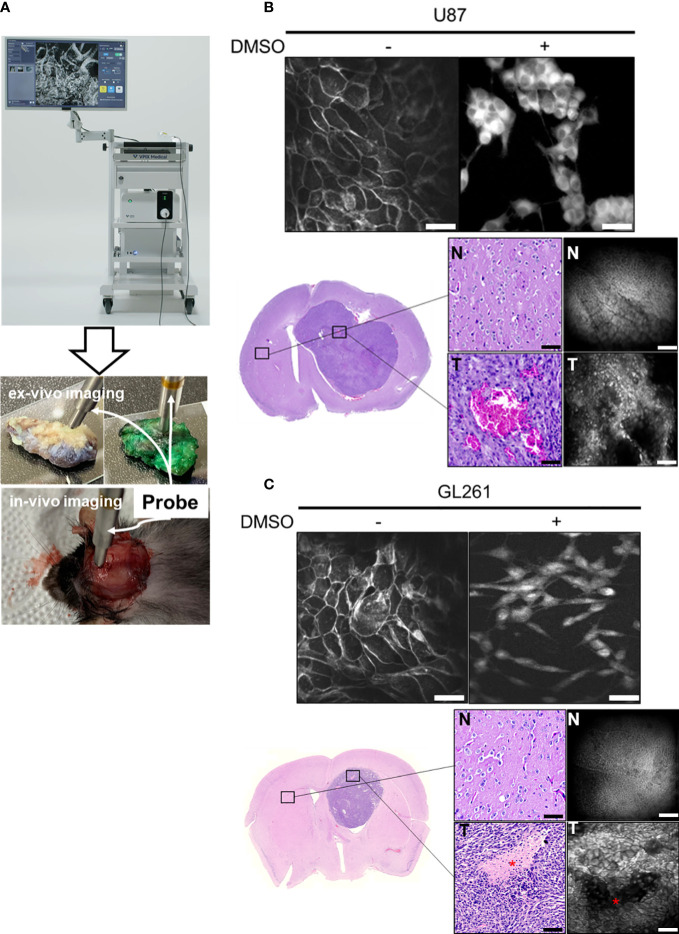
CLE systems and photomicrographs of various glioma models **(A)** The Lissajous scanning CLE system consists of an imaging screen, miniaturized probe, and excitation light source. The probe can detect the CLE images of brain tumor tissue both *ex vivo* and *in vivo*. In the current study, only *ex-vivo* imaging was obtained and compared. **(B, C)** Image acquisition of U87 human and GL261 mouse glioma cells was conducted using CLE following incubation with 5 mg/mL indocyanine green (ICG) (upper panel). Cell morphology was examined in the presence/absence of dimethyl sulfoxide (DMSO). In an orthotopic xenograft model, ICG-induced fluorescent imaging and the corresponding H&E stain were obtained at the tumor core and normal brain (lower panel). CLE image shows dense fluorescent neoplastic cells following ICG incubation *ex vivo*. N: normal brain, T: tumor, Asterix: necrosis. Scale bar: 50 µm. H&E = hematoxylin and eosin.

Using the ICG dye on a handheld CLE with special laser scanning called “Lissajous pattern” (cCell, VPIX Medical) (**Figure 2A**), we first verified the CLE images against U87 and GL261 glioma cell lines in the presence or absence of DMSO. Interestingly, the *in vitro* CLE images showed different patterns depending on the presence of DMSO. In the absence of DMSO, ICG could not penetrate the cells, resulting in a mesh-like shape, in which only the cell membrane was stained. In contrast, in the presence of DMSO, ICG penetrated the cell, and CLE images demonstrated a morphology that is similar to fluorescence immunocytochemical staining, which identifies the nucleus and cell membrane (**Figures 2B, C**). To reproduce the clinical conditions of intraoperative tumor tissue diagnosis, we compared *ex vivo* CLE images with pathological H&E staining using an orthotopic glioma model. U87 human and GL261 mouse glioma cells (1 × 10^5^ cells) were implanted into the brain parenchyma of BALB/c nude mice and C57BL/6 mice, respectively. The mouse brains were removed and cut horizontally, and CLE and H&E staining were performed on the same tissue specimens. The U87 xenograft and GL261 syngeneic gliomas showed variegated and large tumor cells with increased cellularity in the core region, whereas normal brain tissues were found to be relatively homogeneous with sparse cell density (**Figures 2B, C**). In addition, necrotic areas were observed in both the CLE images and H&E staining (**Figure 2C**).

#### 3.1.2 Tumor invasion at patient-derived xenograft model

**Figure 3:**
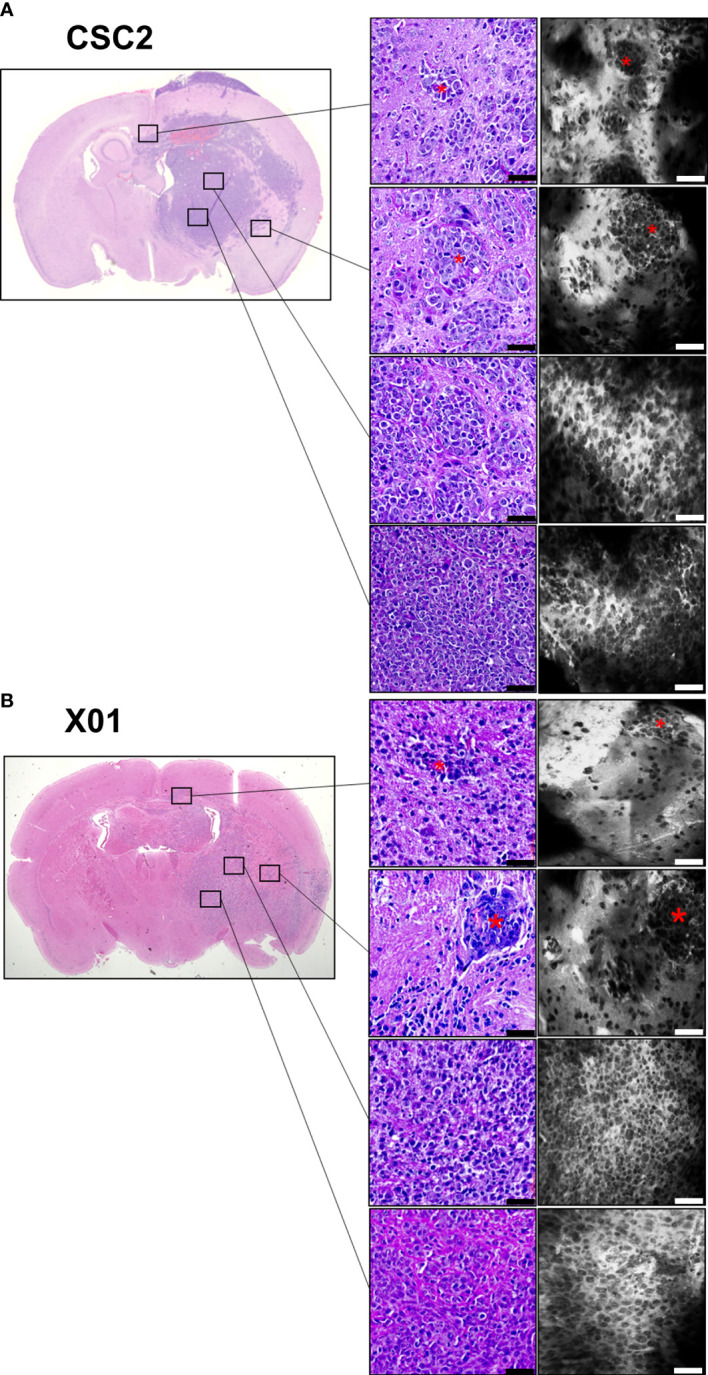
Photomicrographs in patient-derived orthotopic xenograft models. CSC2 **(A)** and X01 **(B)** patient-derived glioma stem-like cells (1 × 10^5^) were injected into BALB/c nude mouse brains. Six weeks later, the mouse brain was obtained and incubated with indocyanine green fluorescent dye. CLE images and H&E staining of the same tissues demonstrated two different glioma tissue cores and peripheral tumor margins, respectively. Asterix: Invasive cells. Scale bar: 50 µm. H&E, hematoxylin and eosin.

We used an orthotopic mouse model with patient-derived glioma cells to identify tumor invasion adjacent to the normal brain tissue. CSC2 and X01 cells (1×10^5^ cells) were implanted into the brain parenchyma of BALB/c nude mice. After 42 days, CLE imaging was performed using the strategy shown in **Figure 2B**. Surprisingly, at multiple different regions, invasive tumor cells were observed in the dense cell cluster adjacent to normal tissue borders in both CSC2 and X01 orthotopic gliomas (**Figures 3A, B** and **Supplementary Video 1**). The tissue morphology and tumor cell infiltration in the CLE images were similar to those observed by histological staining. CLE images demonstrated the same morphological features in CSC2 glioma tissue when ICG was incubated or injected into the tail vein (**Supplementary Figure 1**).

### 3.2 Surgically resected human brain tumor tissues

A total of 69 patients were prospectively enrolled in this study, with a mean age of 56.06 years (range: 20–84 years). The detailed tumor subtypes are listed in **Table 1**.

**Table 1:** Histopathologic diagnosis of 69 human brain tumor samples.

Diagnosis	Histopathologic subtype	Number of cases
**Glioma**	WHO Grade 2	4
	WHO Grade 3	5
	WHO Grade 4	11
**Meningioma**	Meningothelial	10
	Transitional	9
	Psammomatous	1
	Atypical	2
**Metastatic tumor**	Adenocarcinoma	4
	Squamous cell carcinoma	2
	Melanoma	2
	Others	3
**Pituitary adenoma**		11
**Schwannoma**		2
**Primary CNS lymphoma**	Diffuse large B cell	1
**Solitary fibrous tumor**		1
**Choroid plexus papilloma**		1

WHO, World Health Organization; CNS, central nervous system.

#### 3.2.1 CLE images and histology in normal brain parenchyma

**Figure 4:**
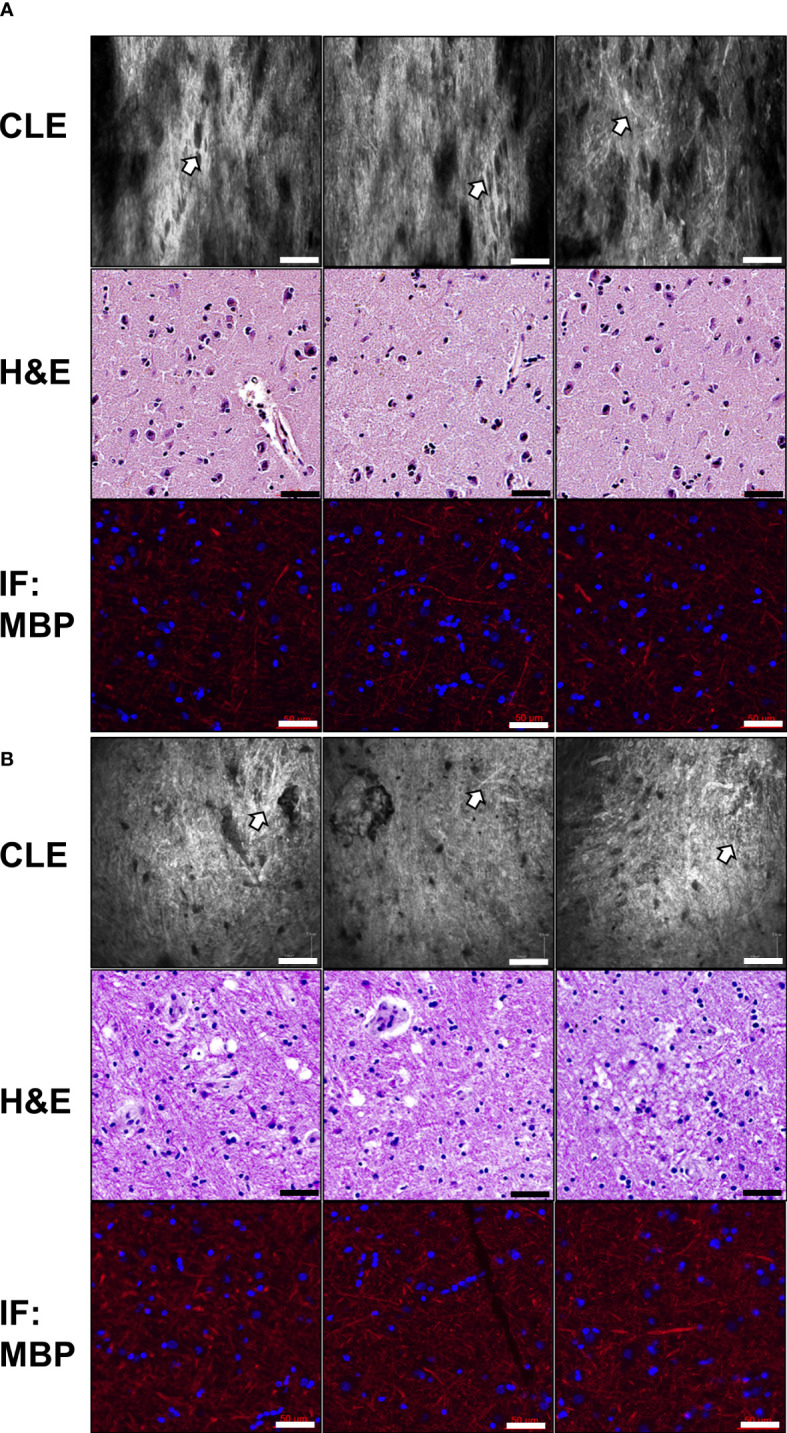
Representative images of normal human brain tissues Normal brain tissues were obtained from the cerebral cortex **(A)** and subcortical areas **(B)** during surgical resection. CLE images show a dense streak-like pattern throughout most of the brain mass (white arrows). H&E staining revealed the presence of large neuronal cells **(A)** and glial cells **(B)**. Myelin basic protein (MBP) immunostaining of the same tissue sample revealed dense axonal fibers (red). Scale bar: 50 µm. H&E, hematoxylin and eosin.

To identify normal human brain morphology in the CLE images, three normal brain tissues were obtained from regions adjacent to the resected intraparenchymal tumors. ICG (5 mg/mL) incubation was performed after surgical resection, and normal brain specimens were examined *ex vivo* using CLE. To obtain consistent image data, at least 100 images were acquired at multiple locations per tissue sample (**Figure 4**). In the gray matter, large neuronal cells were identified in the CLE images, similar to those observed with H&E staining (**Figure 4A**). Compared with the cortical region, glial cells were smaller in size in the subcortical white matter, and a dense streak-like pattern was more frequently observed in the extracellular space, suggesting the presence of myelin fibers (**Figure 4B**). IF staining of MBP revealed myelination of the brain parenchyma in both the gray and white matter. No autofluorescence was observed in the CLE images when ICG incubation was not performed (data not shown).

#### 3.2.2 Pathognomonic CLE findings according to tumor subtypes

**Figure 5:**
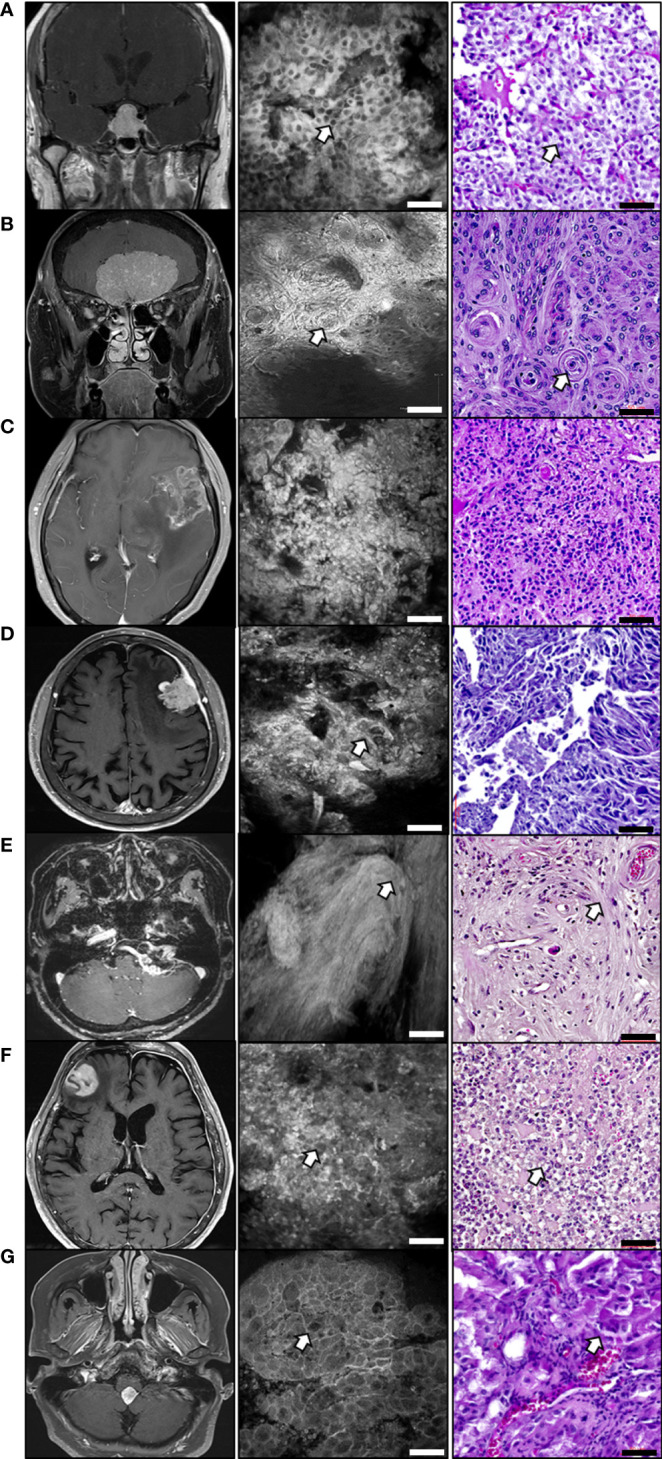
*Ex vivo* CLE images compared to histology in various brain tumor tissues Corresponding representative images of brain tumors consist of T1 enhanced magnetic resonance images (left), CLE images (middle), and H&E staining (right). **(A)** Pituitary adenoma. White arrows indicate the salt-and-pepper patterns. **(B)** Olfactory groove meningioma. White arrows indicate a sheet or lobular architecture that contains a whorl pattern. **(C)** Glioblastoma image showing the overall hypercellularity and atypia. **(D)** Metastatic adenocarcinoma. White arrows indicate atypical cell nests. **(E)** Vestibular schwannoma. White arrows indicate the spiral pattern of whorl formation. **(F)** Primary central nervous system lymphoma. White arrows indicate the large, atypical lymphocytes. **(G)** Choroidal plexus papilloma. White arrows indicate papillary structures lined with uniform cuboidal or columnar epithelial cells. Scale bar: 50 µm. H&E, hematoxylin and eosin.

We examined the concordance between CLE images and histological features using H&E staining of patient tumor tissues. As shown in **Figure 5** and **Supplementary Figures 2–4**, various brain tumors have distinct tissue microstructures. The pituitary adenoma showed a salt-and-pepper pattern, in which tumor cells had monomorphic nuclei and granular cytoplasm in histology and CLE images (**Figure 5A** and **Supplementary Video 2**). In meningiomas, pathognomonic features of the meningothelial type were observed in both images. Meningiomas, which had unclear cell borders and abundant cytoplasm, demonstrated a sheet or lobular pattern (whorl formation) (**Figure 5B**, **Supplementary Figure 2B** and **Supplementary Video 3**). The CLE and H&E images of the glioblastoma showed overall hypercellularity and atypical features (**Figure 5C** and **Supplementary Figure 3B**). Metastatic brain adenocarcinoma also showed a similar pattern in both images, demonstrating atypical cell nests or cord-like structures (**Figure 5D** and **Supplementary Figure 3C**). In vestibular schwannoma, low cellularity and a spiral whorl formation were observed in the H&E images, and similar morphological features were also revealed in the CLE images (**Figure 5E** and **Supplementary Figure 2C**). Primary central nervous system (CNS) lymphoma had large and atypical basophilic lymphocytes with frequent necrosis in both images (**Figure 5F** and **Supplementary Figure 4A**). Histological examination of the choroid plexus papilloma revealed tumor cells containing papillary structures lined by uniform cuboidal or columnar epithelial cells. In CLE images, globular cauliflower-like masses were formed by cuboidal to columnar cells (**Figure 5G** and **Supplementary Figure 4B**).

**Figure 6:**
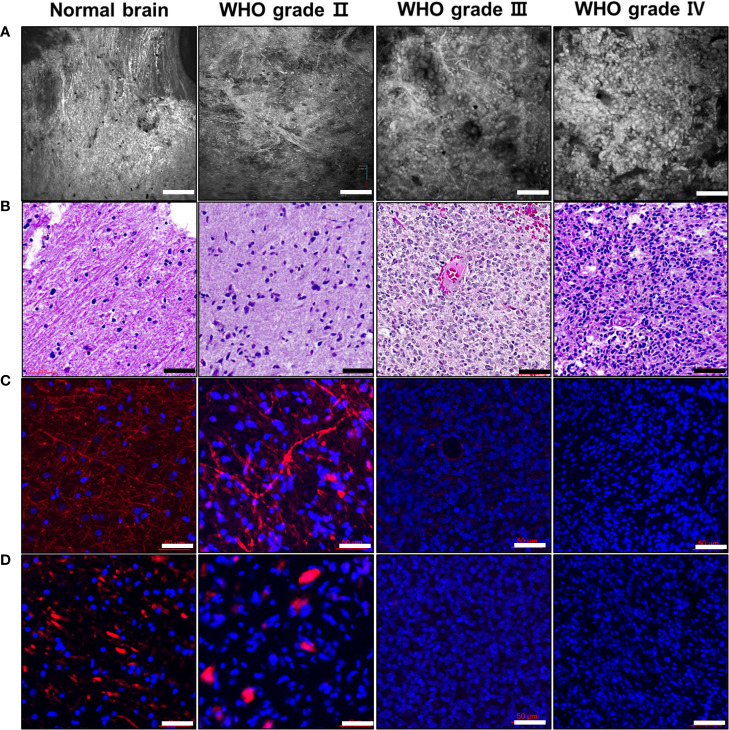
Representative photomicrographs of CLE images and glioma histology Compared to normal brain tissue, gliomas are classified as *IDH1*-mutant gliomas (WHO grade II), anaplastic oligodendroglioma (WHO grade III), and glioblastoma (WHO grade IV). **(A)** CLE images of different glioma samples, according to their corresponding WHO grades, were obtained. Each glioma sample displayed a variety of morphological phenotypes, including cellularity and streak-like patterns. **(B)** H&E staining of the same tissue showing distinct histological differences in glioma grade, which was examined to identify cellularity, atypia, mitosis, and angiogenesis. **(C, D)** As a typical marker for axon fibers, myelin basic protein (MBP) and neurofilament stains were examined at the corresponding glioma tissues. Similar to the CLE images, the amount of MBP and neurofilament staining were significantly decreased in high-grade gliomas. Scale bar: 50 µm. H&E, hematoxylin and eosin; WHO, World Health Organization.

Gliomas originate from glial cells in the brain parenchyma and are classified into different grades based on tumor aggressiveness. To identify whether the tumor grade could be divided into CLE images, we compared the histology with CLE images of normal brain tissues and different gliomas (**Figure 6**). In the normal brain, glial cells were sparsely distributed on H&E staining, and dense axonal fibers were identified in the IF image of MBP and neurofilaments that exhibited CNS myelination. In addition, dense streak-like axonal patterns were observed in CLE images. In gliomas, tumor cellularity was positively associated with tumor grade on histology and CLE images. In particular, high-grade gliomas had significantly increased cellularity, and the tumor cell distribution was dysmorphic and heterogeneous. Conversely, the amount of MBP and neurofilaments was lower in high-grade gliomas than in low-grade gliomas in IF staining. This pattern also revealed CLE images, in which streak-like patterns were rarely identified in high-grade gliomas.

#### 3.2.3 Panoramic CLE images with 3D depth information

**Figure 7:**
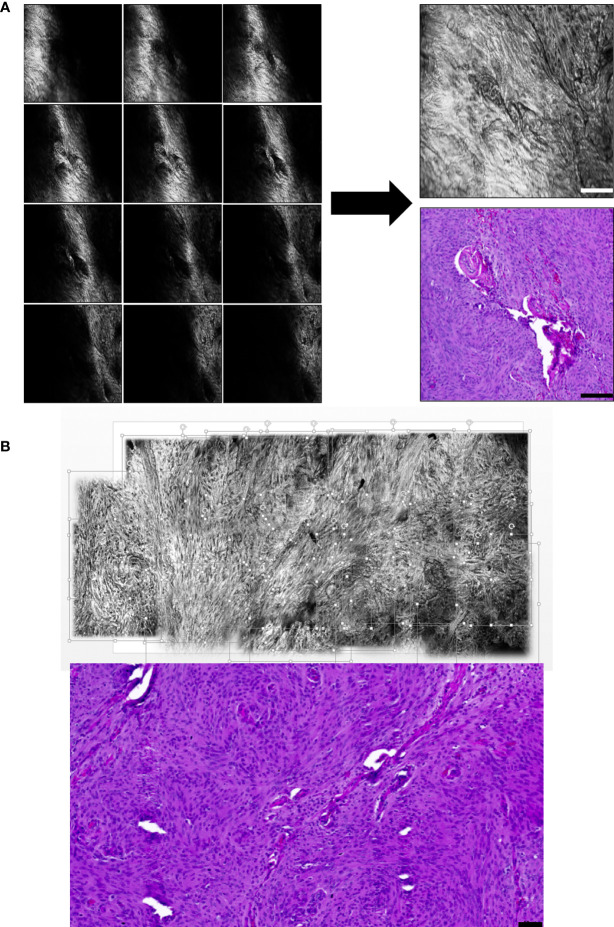
Panoramic CLE images with 3D depth information and their comparison with conventional hematoxylin and eosin stain A series of images taken while changing the distance between the probe and the tissue in the Z-axis were merged into one image (i.e., Z-merged). **(A)** The Z-merged CLE image and the histology of the benign meningioma tissue show similar microstructural features. **(B)** Using meningioma tissue, each Z-merged CLE image was combined into a panoramic image to provide a large field of view as much as conventional H&E stain. Scale bar: 50 µm. H&E, hematoxylin and eosin.

To obtain panoramic cCeLL images of the brain tumor tissue, two consecutive steps were followed: Z-merge and stitching techniques. We reconstructed CLE images using a meningioma tissue sample. First, CLE images cannot always be used to obtain the whole tissue microstructure per image, because the tumor tissue has a 3D structure (**Figure 7A**, left). To overcome this issue, a series of images of multiple focal planes was captured and merged into one image or Z-merged at each spot with an overlapping area of approximately 30% (**Figure 7A**, right). In addition, the FOV of the CLE images is limited to 300 × 300 µm and does not demonstrate a large tissue area. Therefore, Z-merged images of the tissue were combined or stitched to produce panoramic images with 3D depth information (**Figure 7B**).

## 4 Discussion

In this study, we identified the clinical feasibility of real-time brain tumor diagnosis using the Lissajous scanning CLE. Using an ICG fluorescent dye, various brain tumors showed typical cellular morphologies and distinct tissue microstructures. In addition, CLE clearly identified the normal brain to tumor lesions and revealed tumor cell invasion in the adjacent normal brain. Finally, the Z-merge and stitching techniques overcome the limited FOV acquisition due to tumor tissue irregularity and improve the diagnostic potential.

Handheld CLEs have been successfully developed to detect real-time histologic images in clinical studies (23, 24). In the current study, we used a confocal laser-scanning imaging system based on the Lissajous pattern. It visualizes tissue microstructure using ICG fluorescent dye and obtains high-resolution images of 1024 × 1024 pixels with high frame rates of up to 10 Hz. Unlike other pattern-based micro-laser scanners, Lissajous scanning has a great advantage in that it has a high degree of freedom to adjust the image resolution and frame rate by selecting a driving frequency in a pseudo-resonant frequency range that realizes a satisfactory scanning amplitude (25). This feature allows our system to be used in a mode capable of high-speed imaging when operated with a handheld and high-resolution mode when used with an auto-stage capable of precise movement.

In our study, brain tissue visualization *via* CLE imaging allowed for the distinction of brain tumors based on their unique morphological features (26). The CLE images closely resembled the H&E-stained histological images. It is well known that tumor tissues can disrupt normal extracellular composition (27), increase cellularity (28), and display unique brain tumor phenotypic morphologies, such as the presence of psammoma bodies in meningioma tissues (29). In our CLE and histologic images, there were synchronous typical cell morphologies, such as salt-and-pepper patterns in pituitary adenoma and uniform cuboidal or columnar epithelial cells in choroid plexus papilloma. In addition, other tissue microstructures were also found, including whorl formation in meningiomas, atypical cell nests in metastatic adenocarcinomas, and spiral patterns in schwannomas. Furthermore, some tumor cells, such as pituitary adenoma and choroid plexus papilloma, revealed intracellular morphology, but ICG could not penetrate the cell membrane in U87 glioma cells. It has been suggested that intracellular ICG uptake correlates with endocytosis potential and tight junctions, although the mechanism of intracellular ICG uptake remains unclear (30). The CLE images not only increased the diagnostic potential but also significantly shortened the time to reach a precise diagnosis. For instance, in the current study, the mean operation time to obtain a CLE image was less than one second per frame. Considering that seven *ex vivo* images were acquired before the identification of the first brain tumor diagnostic image, real-time diagnosis could be performed intraoperatively (11).

Maximal surgical resection of glial tumors is critical for patient survival and recurrence rates (31–33). Current methods of tumor detection, including a neuronavigation system and intraoperative visualization *via* 5-ALA, have improved tumor resection (5, 6). However, there are some limitations, including brain shifts and variable fluorescence in the glioma tissues (34). In addition, glioma cell invasion into the normal brain is not determined during intraoperative neuronavigation or macroscopic 5-ALA tumor fluorescence. Recently, fluorescein sodium (FNa) was used as a fluorescent agent for glioma resection during intraoperative glioma visualization. Intravenously injected FNa selectively accumulates in the brain and can pass through an altered blood-brain barrier (BBB). This selectivity provides the ability of FNa to be used as a guide during fluorescein-guided resection of gliomas (35, 36). However, FNa is also not specific and can accumulate in some peritumoral areas, leading to reduced accuracy in terms of tumor identification (37). It has been suggested that direct tumor cell visualization can overcome these issues. CLE is a useful tool for glioma diagnosis in the neurosurgical field. Fluorescent agents, including FNa and ICG, have been used to detect cytoarchitectures of normal and glioma tissues (38, 39). In the current study, we developed an orthotopic intracranial model using patient-derived glioma cells that were histologically identical to human glioma tissue (40). In the CLE images, heterogeneous and hypercellular regions were found at the tumor core and infiltrative glioma cell clusters were observed at the edge of the glioma. These features are identical to those observed in the histological images. In addition, obtaining CLE images and determining whether the ICG fluorescence dye was injected into the vein or incubated with glioma tissue *ex vivo* were not critical issues in our study (**Supplementary Figure 1**).

In the CLE images, we easily identified the differences between normal brain and glioma tissues. Compared to the normal brain, gliomas revealed hypercellularity and dysmorphic morphological features that were positively associated with tumor grade. Interestingly, CLE imaging of normal brain and low-grade glioma tissues demonstrated a distinct streak-like pattern throughout most of the tissue areas. To determine whether these streak-like patterns represent myelin fibers, MBP and neurofilament staining were performed in one normal brain tissue and three different glioma types: *IDH1*-mutant glioma, anaplastic oligodendroglioma, and glioblastoma. The density of the streak-like pattern in CLE images was strongly correlated with the amount of MBP and neurofilament in the normal brain and gliomas, and there was a reverse correlation between glioma grade and the quantity of these myelin fibers. Therefore, streak-like patterns can be utilized as cues to identify glioma grade in CLE images.

The FOV of a CLE image is limited by the scanning amplitude of the micro-laser scanner. Given that a micro-laser scanner needs to come into contact with the patient’s body, it is difficult to acquire large-area images simultaneously because high voltages and currents are required for this process. Therefore, most commercially available CLEs generally have a narrow FOV of 240 × 240 µm to 600 × 500 µm (41). In addition, CLE images are obtained from black areas outside the focus because the tumor tissue has a 3D structure, and the image signal is blocked because of the high axial resolution. These drawbacks can be overcome with stitching or mosaicing techniques that connect the acquired images *via* the Z-merge function, which moves the probe in the depth direction to acquire tissue images along different Z-axes and merges the images into one image (17). In this study, a panoramic image was obtained by connecting the Z-merged images horizontally and vertically (**Figure 7B**). It took approximately 10 min to manually connect the 20 images. If a real-time panoramic function based on an automatic stitching algorithm is developed, its clinical usefulness is expected to increase.

On summary, a newly developed CLE device can be equipped in operation settings and easily utilized with miniaturized hand-held probe. It can visualize the cytoarchitecture of the fresh or resected human tissue including brain and other organs. It is potentially safe because it does not contact the tissue and we do not need to resect the tissue for histologic confirmation. Further panoramic view reconstruction and pseudo-coloring effects are now being exploited. After verifying the diagnosis accuracy for brain tumor subtype, we hope it can substitute the frozen tissue histology performed by pathologist outside the operation room.

The current study had several limitations. First, it was designed as an observational study that focused on verifying the feasibility of a new CLE device, so the *ex vivo* brain tumor samples were inhomogenously included as electively scheduled. Second, a glioma cell line only exists in experimental *in vitro* brain tumor settings, and other subtypes of brain tumor were not observed. To overcome these limitations, we are currently conducting a multicenter clinical prospective study to identify the diagnostic accuracy of the device in depth. Third, the current findings were obtained from a single center and multicentered study design is necessary for the device application. Finally, the current study was designed as an observational study and did not include statistical analysis. Again, a future multicenter clinical trial with statistical analysis is needed.

## Data availability statement

The original contributions presented in the study are included in the article/**Supplementary Material**. Further inquiries can be directed to the corresponding author.

## Ethics statement

The studies involving human participants were reviewed and approved by Institutional Review Board of Korea University Anam Hospital. The patients/participants provided their written informed consent to participate in this study. The animal study was reviewed and approved by Institutional Animal Care and Use Committee of Korea University College of Medicine. Written informed consent was obtained from the individual(s) for the publication of any potentially identifiable images or data included in this article.

## Author contributions

DH and JK designed and performed most of the experiments, analyzed the data, and wrote the manuscript. CK, KH, and K-HJ performed CLE imaging and wrote the manuscript. HK helped design and perform the animal experiments. K-JP helped in writing the manuscript. J-KW provided the pathologist’s expertise in comparing the CLE images to the H&E histological images. S-HK helped to design the experiments, co-wrote the manuscript, and provided an overall direction.

## Supplementary material

Supplementary Video 1 Real-time examination of the implanted patient-derived glioma tissue (CSC2) from BALB/c nude mouse brain. The H&E stained, and CLE scanned images from the same mouse were presented in **Figure 3A**.

Supplementary Video 2 Real-time examination of the pituitary adenoma tissue from resected human specimen. The H&E stained, and CLE scanned images from the same tissue were presented in **Figure 5A** and **Supplementary Figure 2A**.

Supplementary Video 3 Real-time examination of the meningothelial meningioma tissue from resected human specimen. The H&E stained, and CLE scanned images from the same tissue were presented in **Figure 5B** and **Supplementary Figure 3B**.
