# Corrigendum: Clinical feasibility of miniaturized Lissajous scanning confocal laser endomicroscopy for indocyanine green-enhanced brain tumor diagnosis

**DOI:** 10.3389/fonc.2024.1375620

**Published:** 2024-02-13

**Authors:** Duk Hyun Hong, Jang Hun Kim, Jae-Kyung Won, Hyungsin Kim, Chayeon Kim, Kyung-Jae Park, Kyungmin Hwang, Ki-Hun Jeong, Shin-Hyuk Kang

**Affiliations:** ^1^ Department of Neurosurgery, Korea University Hospital, Korea University College of Medicine, Seoul, Republic of Korea; ^2^ Department of Pathology, Seoul National University Hospital, Seoul National University College of Medicine, Seoul, Republic of Korea; ^3^ VPIX Medical Inc., Daejeon, Republic of Korea; ^4^ Department of Bio and Brain Engineering, KAIST Institute for Health Science and Technology (KIHST), Korea Advanced Institute of Science and Technology (KAIST), Seoul, Republic of Korea

**Keywords:** brain neoplasm, confocal microscopy, Lissajous scanning, indocyanine green, real-time diagnosis

In the published article, there was an error in the Funding statement. The funding statement for the Korea Health Technology R&D Project through the Korea Health Industry Development Institute (KHIDI), funded by the Ministry of Health & Welfare, Republic of Korea was displayed as “HI22C1302”. The correct statement is “Korea Health Technology R&D Project through the Korea Health Industry Development Institute (KHIDI), funded by the Ministry of Health & Welfare, Republic of Korea (grant number: HR22C1302)”. The correct complete Funding statement appears below.

**This research was supported by a grant from the Korea Health Technology R&D Project through the Korea Health Industry Development Institute (KHIDI), funded by the Ministry of Health & Welfare, Republic of Korea (grant number: HR22C1302), and by the Korea Medical Device Development Fund grant funded by the Korea government (the Ministry of Science and ICT, the Ministry of Trade, Industry and Energy, the Ministry of Health & Welfare, the Ministry of Food and Drug Safety) (grant number: RS-2022-00197971).**

The authors apologize for this error and state that this does not change the scientific conclusions of the article in any way. The original article has been updated.

