# Supplementary material for: Clinical feasibility of miniaturized Lissajous scanning confocal laser endomicroscopy for indocyanine green-enhanced brain tumor diagnosis
Source: Front Oncol. 2023 Jan 13;12:994054. doi: 10.3389/fonc.2022.994054 (PMC9880156; doi:10.3389/fonc.2022.994054)
Supplement: Supplementary file 1 [file Table_1.docx]

**
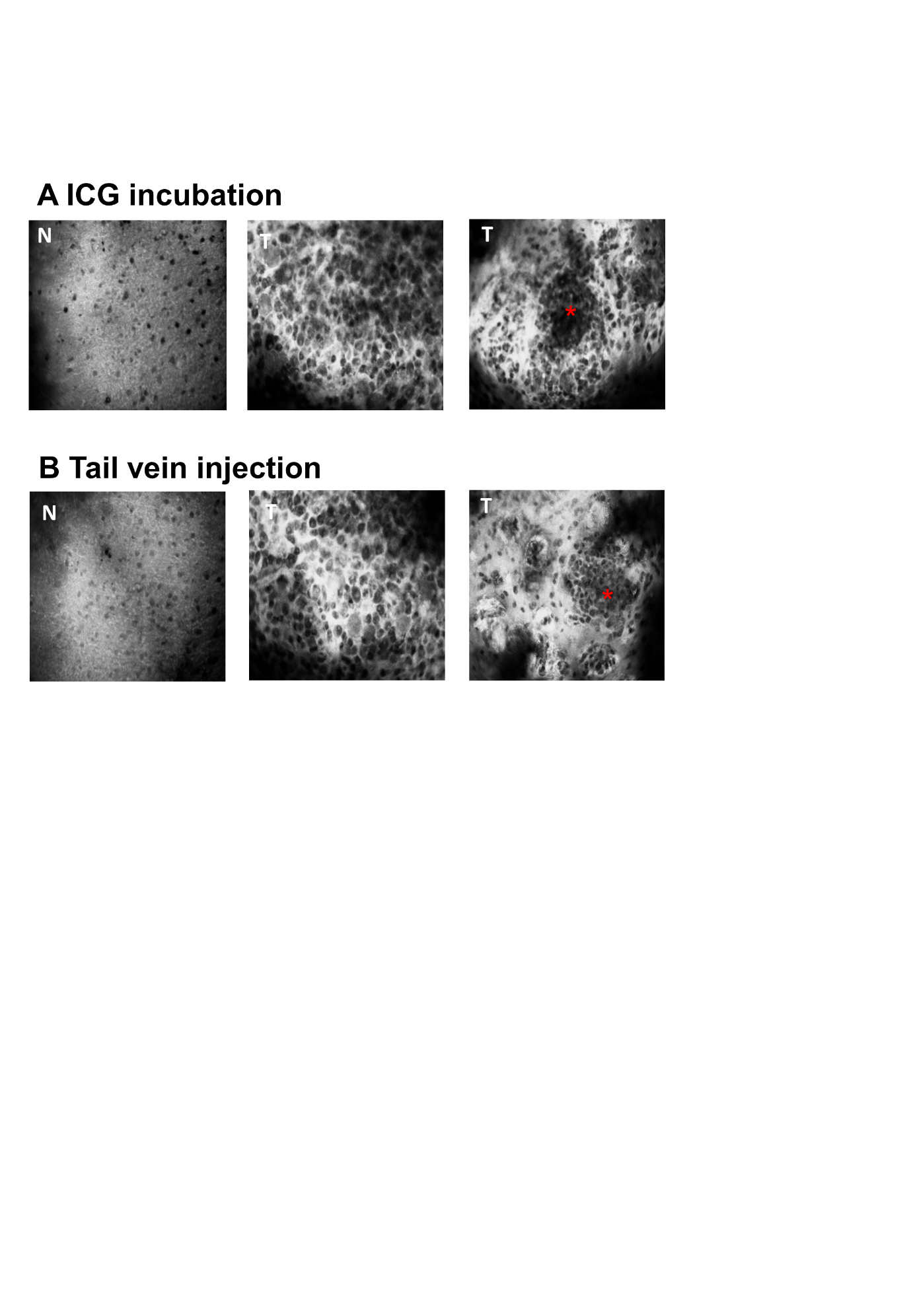
**

**Supplementary Figure 1.** CLE images depending on ICG incubation or tail vein injection in CSC2 glioma intracranial model. Asterix represents glioma invasion to the normal brain. N= normal brain, T= tumor

**
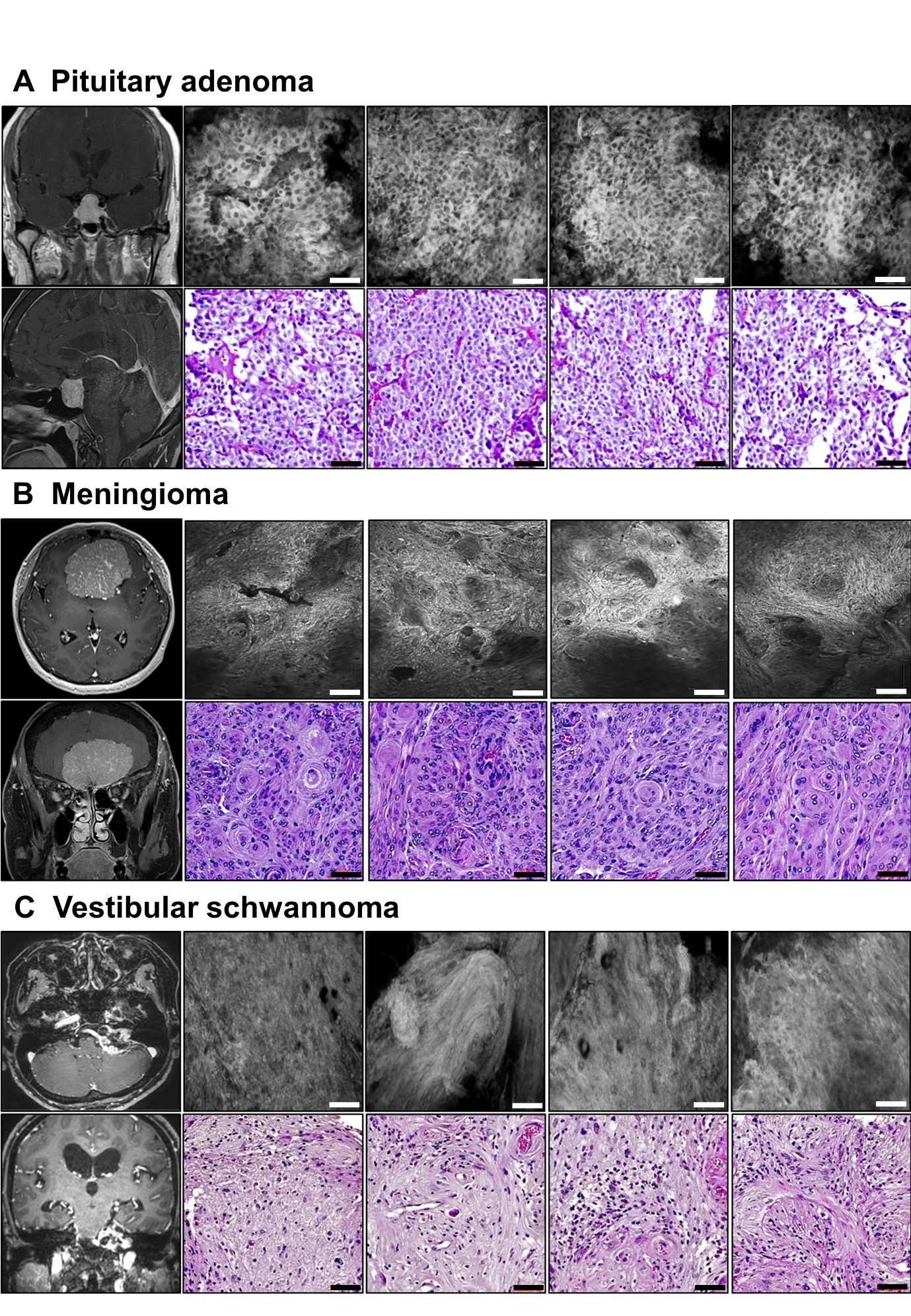
**

**Supplementary Figure 2.** Representative MR images, patient matched CLE and histologic images of different tumor regions in pituitary adenoma, meningioma and glioblastoma

**
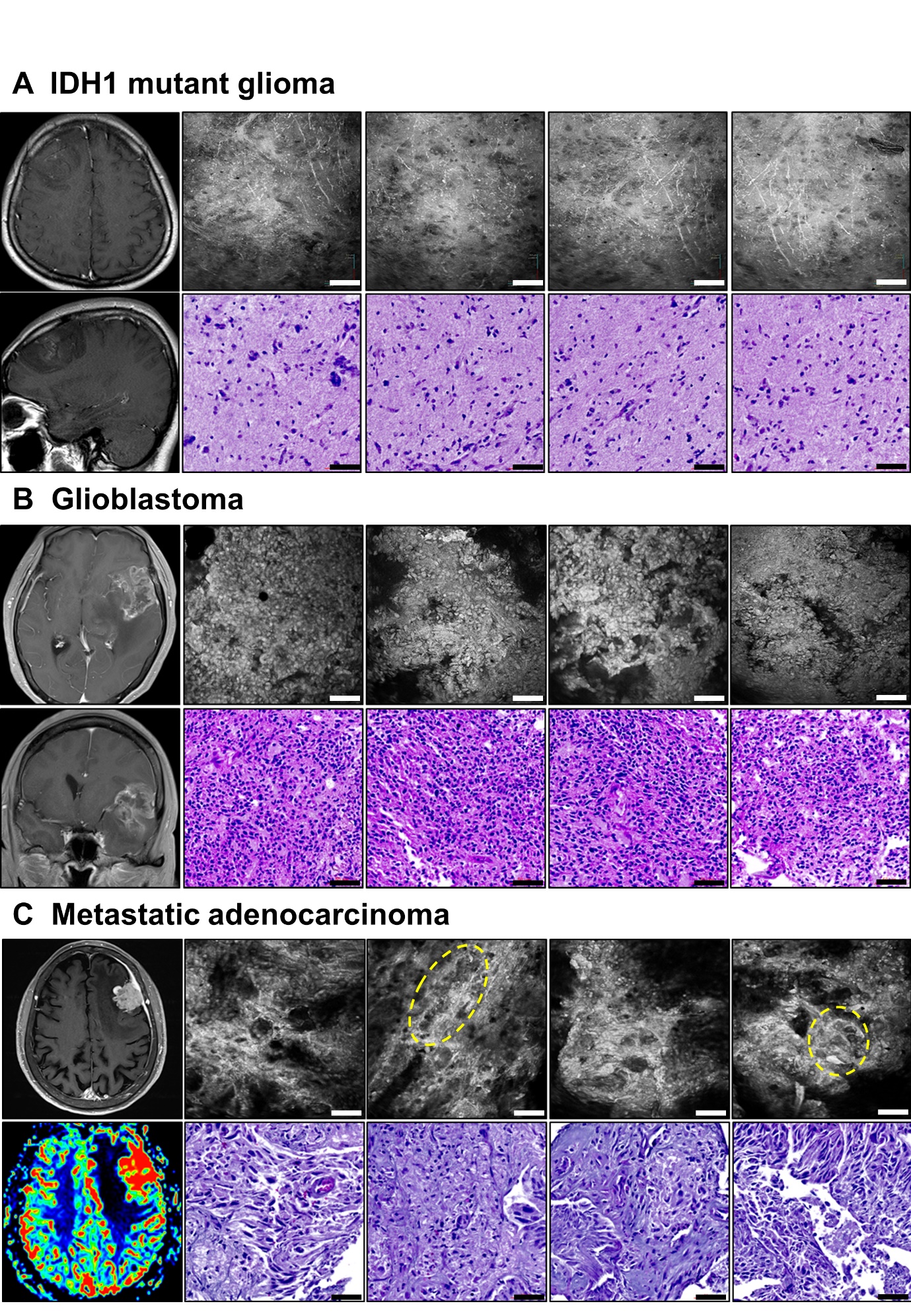
Supplementary Figure 3.** Representative MR images, patient matched CLE and histologic images of different tumor regions in metastatic adenocarcinoma and vestibular schwannoma

**
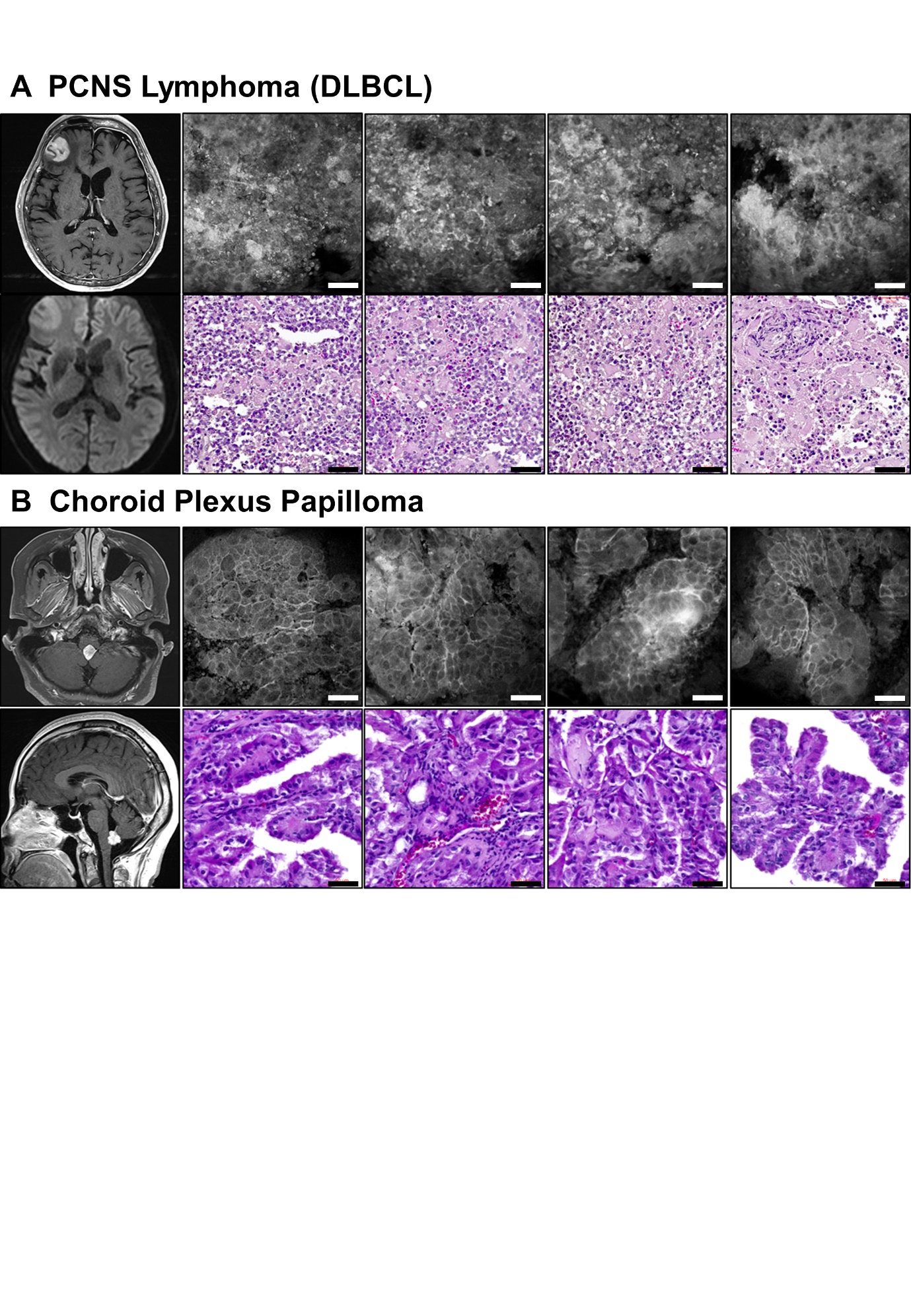
**

**Supplementary Figure 4.** Representative MR images, patient matched CLE and histologic images of different tumor regions in primary CNS lymphoma and choroid plexus papilloma
